# Predicting grade II-IV bone marrow suppression in patients with cervical cancer based on radiomics and dosiomics

**DOI:** 10.3389/fonc.2024.1493926

**Published:** 2024-11-28

**Authors:** Yanchun Tang, Yaru Pang, Jingyi Tang, Xinchen Sun, Peipei Wang, Jinkai Li

**Affiliations:** ^1^ Department of Radiation Oncology, The First Affiliated Hospital of Nanjing Medical University, Nanjing, Jiangsu, China; ^2^ The First Affiliated Hospital of Nanjing Medical University, Nanjing Medical University, Nanjing, Jiangsu, China

**Keywords:** cervical cancer, bone marrow suppression, radiomics, dosiomics, machine learning

## Abstract

**Objective:**

The objective of this study is to develop a machine learning model integrating clinical characteristics with radiomics and dosiomics data, aiming to assess their predictive utility in anticipating grade 2 or higher BMS occurrences in cervical cancer patients undergoing radiotherapy.

**Methods:**

A retrospective analysis was conducted on the clinical data, planning CT images, and radiotherapy planning documents of 106 cervical cancer patients who underwent radiotherapy at our hospital. The patients were randomly divided into training set and test set in an 8:2 ratio. The radiomic features and dosiomic features were extracted from the pelvic bone marrow (PBM) of planning CT images and radiotherapy planning documents, and the least absolute shrinkage and selection operator (LASSO) algorithm was employed to identify the best predictive characteristics. Subsequently, the dosiomic score (D-score) and the radiomic score (R-score) was calculated. Clinical predictors were identified through both univariate and multivariate logistic regression analysis. Predictive models were constructed by intergrating clinical predictors with DVH parameters, combining DVH parameters and R-score with clinical predictors, and amalgamating clinical predictors with both D-score and R-score. The predictive model’s efficacy was assessed by plotting the receiver operating characteristic (ROC) curve and evaluating its performance through the area under the ROC curve (AUC), the calibration curve, and decision curve analysis (DCA).

**Results:**

Seven radiomic features and eight dosiomic features exhibited a strong correlation with the occurrence of BMS. Through univariate and multivariate logistic regression analyses, age, planning target volume (PTV) size and chemotherapy were identified as clinical predictors. The AUC values for the training and test sets were 0.751 and 0.743, respectively, surpassing those of clinical DVH R-score model (AUC=0.707 and 0.679) and clinical DVH model (AUC=0.650 and 0.638). Furthermore, the analysis of both the calibration and the DCA suggested that the combined model provided superior calibration and demonstrated a higher net clinical benefit.

**Conclusion:**

The combined model is of high diagnostic value in predicting the occurrence of BMS in patients with cervical cancer during radiotherapy.

## 1 Introduction

Cervical cancer stands as a leading gynecological malignancy worldwide, occupying the fourth position in cancer-related deaths among women. Currently, the primary approach to treating cervical cancer is a comprehensive strategy centered on surgical intervention. However, for patients who are ineligible for surgery or face a risk of recurrence post-operatively, radiotherapy serves as an effective adjunctive treatment option. But radiotherapy can bring toxic side effects to different degrees. The most prevalent among these is bone marrow suppression (BMS), characterized by a significant decrease in leukocyte, neutrophils, hemoglobin, platelets. And the clinical manifestations were anemia, hemorrhage, infection and so on, so severe BMS may delay or interrupt treatment, potentially compromising its effectiveness and exacerbating patients’ condition (1). Therefore, it is critical to identify the factors associated with BMS in cervical cancer patients undergoing radiotherapy and to implement timely interventions.

Previous studies have leveraged general clinical characteristics and dose-volume histogram (DVH) metrics to predict BMS (2). Kumar T et al. found that DVH metrics, including V5, V15 and V20, exhibit a correlation with the occurrence of BMS (3, 4). However, these dosimetrics offer limited insights into three-dimensional (3D) dose distribution and lack spatial context. In the era of big data, dosiomics advances our understanding by thoroughly analyzing dose distribution files, extracting multi-dimensional information such as texture, morphology, shape, etc. (5, 6) In addition, considering heterogeneity among patients, it is essential to tailor BMS predictions to reflect the unique bone marrow characteristics of each individual.

On the other hand, radiomics is a non-invasive, reliable and intuitive diagnostic method that can transform images into a large number of quantitative features (7). MRI radiomics have now been shown to be valuable in predicting the radiotherapy response of patients with cervical cancer patients (8). Previous studies have demonstrated that combining dosiomics with radiomics can improve the prediction of radiation pneumonitis (9, 10). However, there is no research on the use of radiomics and dosiomics to explore the side effects of radiotherapy in patients with cervical cancer. Inspired by them, we developed a comprehensive predictive model of the occurrence of BMS in patients with cervical cancer.

Therefore, the purpose of this study was to explore the predictive value of radiomics and dosiomics on the occurrence of BMS in cervical cancer patients during radiotherapy based on patient planning CT images and dose distributions files, so as to find out the high-risk patients with BMS in time and provide reference for the design of clinical and physical planning.

## 2 Patients and methods

### 2.1 Patients

Between October 2017 and January 2023, a retrospective collection of data was conducted for patients diagnosed with cervical cancer and admitted to the Department of Radiotherapy at the First Affiliated Hospital of Nanjing Medical University, following the ethical approval granted by our institution (ethics id. 2023-SR-882). The inclusion criteria were as follows: (1) normal blood routine parameters were observed prior to radiotherapy, without any prior hematologic supportive interventions, such as blood transfusion; (2) no history of tumors; and (3) undergoing the first course of chemoradiotherapy. Exclusion criteria: (1) missing hemogram data during radiotherapy; (2) presence of tumors other than cervical cancer; and (3) patients with blood system diseases. Ultimately, 106 patients were included in the study, with 79 receiving concurrent chemotherapy. Patients received concurrent cisplatin chemotherapy in concurrent chemoradiotherapy group, every 4 weeks for 4 cycles. Volume modulated arc therapy (VMAT) and intensity modulated radiation therapy (IMRT) were utilized in radiotherapy. Clinical target volume (CTV) in patients with cervical cancer included vaginal stump, part of the vagina and pelvic lymph node drainage area, with planning target volume (PTV) margin of 0.8cm in all directions. The radiotherapy prescription dose aimed for 95% of target volumes receiving 50Gy in 25 fractions. Patient hemogram indices, i.e. leukocyte, neutrophils, hemoglobin, platelets, were monitored throughout the radiotherapy period. According to the World Health Organization’s classification standards for Bone Marrow Suppression (BMS), a patient’s hemogram indices are classified as Grade 2 or higher, indicating severe BMS, if any of the following criteria are met: leukocyte <3×10^9^/L; neutrophils <1.5×10^9^/L; hemoglobin <95g/L; platelets <75×10^9^/L. For the purpose of assessing the incidence of BMS during radiotherapy treatment, patients were categorized into two groups: those with severe BMS and those with no or mild BMS.

### 2.2 Image pre-processing and region of interest delineation

According to the consensus of RTOG delineation guidelines for female pelvic normal tissues, a gynaecologist delineated the pelvic bone marrow (PBM) on the planning CT images prior to radiotherapy. The delineated PBM encompasses an area extending from the lower edge of L5, and includes the ischium, iliac bone and sacrum. Three files—CT images, PBM, and dose distribution of the patient—were imported into 3D Slicer (version 5.3.0). The dose distribution file was then resampled using the ‘Resample Image’ module to align its resolution with that of the PBM, and subsequently saved in NIfTI (nii) format.

### 2.3 Extraction of radiomic features

The patients were randomly divided into training set and test set according to the ratio of 8:2. The structures of PBM and planning CT images undergo standardization and resampling, with voxel sizes adjusted to 2mm×2mm×2mm. Filtering transformations, such as the Laplacian of Gaussian (LoG) and wavelet, etc., are applied to the images to generate corresponding derived images. Subsequently, a total of 1169 radiomic features for patients with cervical cancer were extracted with Pyradiomics library on these images, including shape features, first-order features, and texture features, etc. (https://pyradiomics.readthedocs.io/en/latest/).

### 2.4 Extraction of dosimetrics and dosiomic features

Similar to radiomics, 1169 dosiomic features were extracted from the dose distribution file of PBMs for each patient. The dosimetrics factors were extracted from the DVH curve of PBM, including the volume fraction of PBM receiving more than 10Gy, 20Gy, 30Gy (V10Gy, V20Gy, V30Gy), as well as the total radiation dose (total dose), mean dose to PBM (Dmean) and the size of PTV.

### 2.5 Feature selection

Character string features were excluded from the radiomic and dosiomic datasets. Features exhibiting significant differences (P < 0.05) were identified using the independent samples t-test. Subsequently, the Least Absolute Shrinkage and Selection Operator (LASSO) algorithm was employed within the training set, utilizing 5-fold cross-validation to refine the selection process. The optimal parameter, λ, was adjusted to minimize the quadratic deviation, thereby identifying the most predictive radiomic and dosiomic features. According to the LASSO regression coefficient, weighted calculation was carried out to obtain the radiomic score (R-score) and dosiomic score (D-score), which were normalized to standardScale. Univariate logistic regression analysis was conducted on the eight clinical characteristics included in this study. These characteristics comprise age, total radiation dose, size of the planned target volume (PTV), whether concurrent chemotherapy was administered, whether surgery was performed, presence of metastasis, pathological type, and the radiotherapy technology utilized. The difference was statistically analyzed by two-sided test. P<0.05 indicated statistically significant, following this, multivariate logistic regression analysis was conducted for the variables to demonstrate statistically significant difference, aiming to identify the clinical characteristics related to the prediction of BMS.

### 2.6 Construction and evaluation of prediction models

A predictive model, termed the Clinical Dosimetrics Prediction Model (Clinics + DVH), was developed utilizing the random forest machine learning algorithm, which integrates clinical characteristics with dosimetric data. Additionally, an enhanced version of this model, the Clinical Dosimetrics R-score Prediction Model (Clinics + DVH + R-score), was also constructed using the same algorithm to incorporate clinical characteristics alongside dosimetric data and R-score. Furthermore, a comprehensive Combined Prediction Model (Clinics + D-score + R-score) was developed, amalgamating clinical characteristics with both R-score and D-score, employing the random forest machine learning algorithm. The efficacy and clinical utility of these models were assessed through various metrics, including the Receiver Operating Characteristic (ROC) curve, the Area Under the Curve (AUC), the calibration curve, and the Decision Curve Analysis (DCA) curve.

### 2.7 Statistical methods

Statistical analyses were conducted using SPSS version 23.0. Continuous variables were analyzed using the t-test, while categorical variables were assessed with the chi-square test. Both univariate and multivariate logistic regression analysis were performed on clinical characteristics with the training set. A two-sided test was used, with a P < 0.05 denoting statistical significance.

## 3 Results

### 3.1 Clinical data

A total of 106 patients were enrolled in this study, including 62 patients in the severe BMS group and 44 patients in the no/mild BMS group. The detailed clinical information of the enrolled patients is presented in **Table 1**. The radiotherapy-alone group consisted of 27 patients, of whom 11 had severe BMS. The concurrent chemoradiotherapy group consisted of 79 patients, of whom 51 had severe BMS. Two groups of data had statistically significant difference (P = 0.03). The age range was 29 to 81. The median age was 49.95 years and 55.24 years for no/mild BMS and severe BMS, respectively, with statistically significant difference (P = 0.02). 73 of 106 patients were irradiated with the VMAT technology, and the remaining 33 were irradiated with IMRT technology. There were significant differences in PTV volumes (P<0.05).

**Table 1:** Patient clinical characteristics [n (%)].

Characteristics		No/mild BMS	Severe BMS	χ^2^/t-value	P -value
Age (y, Mean ± SD)		49.95 ± 12.53	55.24 ± 11.05	-2.295	0.024
Total dose (Gy)		48.43 ± 2.37	48.92 ± 2.65	-0.977	0.331
PTV size (cm3)		1031.27 ± 160.19	1173.08 ± 223.44	-3.602	<0.001
Chemotherapy	No	16 (36.4%)	11 (17.7%)	4.701	0.030
	Yes	28 (63.6%)	51 (82.3%)		
Surgery	No	14 (31.8%)	21 (33.9%)	0.049	0.825
	Yes	30 (68.2%)	41 (66.1%)		
Metastasis	No	31 (70.5%)	37 (59.7%)	1.300	0.254
	Yes	13 (29.5%)	25 (40.3%)		
Pathological type	Squamous carcinoma	41 (93.2%)	53 (85.5%)	1.519	0.218
	Adenocarcinoma	3 (6.8%)	9 (14.5%)		
Radiotherapy techniques	VMAT	31 (70.5%)	42 (67.7%)	0.088	0.766
	IMRT	13 (29.5%)	20 (32.3%)		

BMS, bone marrow suppression; IMRT, intensity modulated radiation therapy; VMAT, volume modulated arc therapy; SD, standard deviation.

### 3.2 Results of feature selection

A total of 1169 radiomic features and dosiomic features were extracted from PBM. 11 radiomic features and 15 dosiomic features were screened by t-test of independent samples. Following this initial selection, feature dimension reduction was achieved by LASSO regression. The super parameter λ, which controls the strength of regularization [a method commonly used for alleviating overfitting in machine learning (11)], was selected by 5-fold cross-validation. It was found that the quadratic deviation of radiomic and dosiomic features was the smallest when λ=0.016 and λ=0.028, in this case, 7 radiomic features and 8 dosiomic features were identify with predictive value. Formula (1), (2) is referenced according to the weight of each feature, the R-score and D-score were calculated and then normalized to standardScale.


(1) $$\begin{array}{c} R-score=0.581081+diagnostics\_Image\\ \;\;\;-interpolated\_Minimum\times (-0.100335)+log\\ \;\;\;-sigma-3-0-mm-3D\_glcm\_Imc1\times \\ \;\;\;(-0.048247)+wavelet-LLH\_firstorder\_Median\times \\ \;\;\;(-0.034577)+wavelet-HLL\_firstoder\_Median\times \\ \;\;\;(-0.39915)+wavelet\\ \;\;\;-HLL\_glrlm\_LongRunLowGrayLevelEmphasis\times \\ \;\;\;(-0.070324)+wavelet\\ \;\;\;-HLL\_glszm\_LowGrayLevelZoneEmphasis\\ \;\;\;\times (0.032038)+wavelet-HHL\_glcm\_Imc1\times \\ \;\;\;(-0.035790) \end{array}$$

(2) $$\begin{array}{l} D-score=0.581081+original\_firstorder\_Kurtosis\\ \;\times (-0.009012)+log-sigma-2-0-mm\\ \;-3D\_glszm\_LargeAreaEmphasis\times (-0.068777)+log\\ \;-sigma-2-0-mm\;\\ \;-3D\_glszm\_LowGrayLevelZoneEmphasis\times (-0.017781)\\ \;+log-sigma-2-0-mm-3D\_glszm\_ZonePercentage\\ \;\times (0.004132)+log-sigma-2-0-mm\\ \;-3D\_gldm\_DependenceEntropy\times (0.034383)+log\\ \;-sigma-3-0-mm-3D\_firstorder\_Skewness\\ \;\times (-0.093013)+wavelet-LHL\_firstorder\_Kurtosis\\ \;\times (-0.076822)+wavelet\\ \;-HHH\_gldm\_DependenceEntropy\times (0.097723) \end{array}$$

Abbreviations: glcm, Gray Level Co-occurence Matrix; glrlm, Gray Level Run-Length Matrix; glszm, Gray Level Size Zone Matrix; gldm, Gray Level Dependence Matrix.

### 3.3 Prediction model construction and evaluation

**Figure 1:**
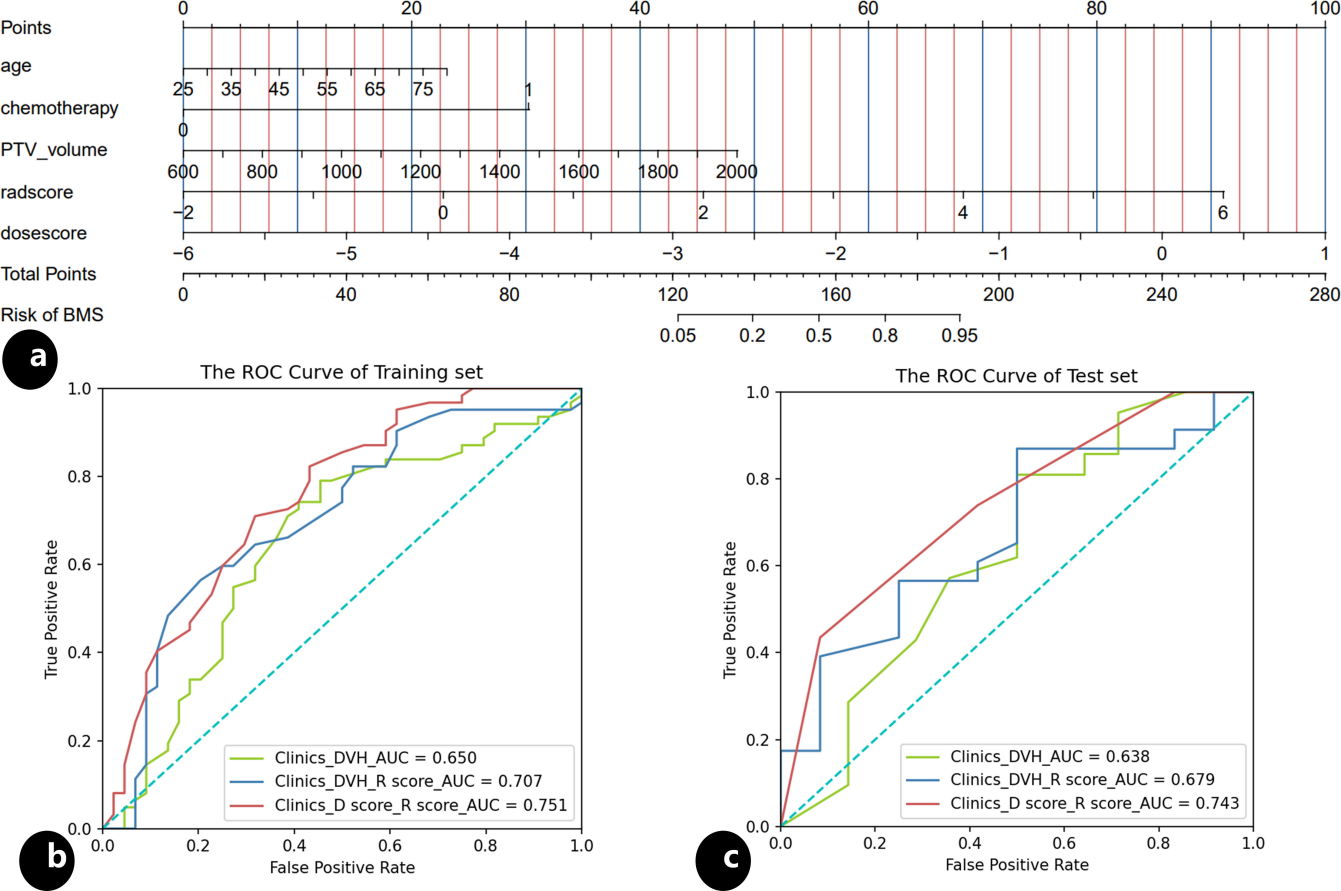
Nomogram and the receiver operating characteristic (ROC) curves, **(A)** nomogram of the Clinics+ D-score+ R-score model predicting occurrence of the BMS during radiotherapy in patients with cervical; **(B)** the ROC for the training set of the Clinics DVH model, the Clinics DVH R-score model and the Clinics+ D-score+ R-score model, respectively; **(C)** the ROC for the test set of the Clinics DVH model, the Clinics DVH R-score model and the Clinics+ D-score+ R-score model, respectively.

**Figure 2:**
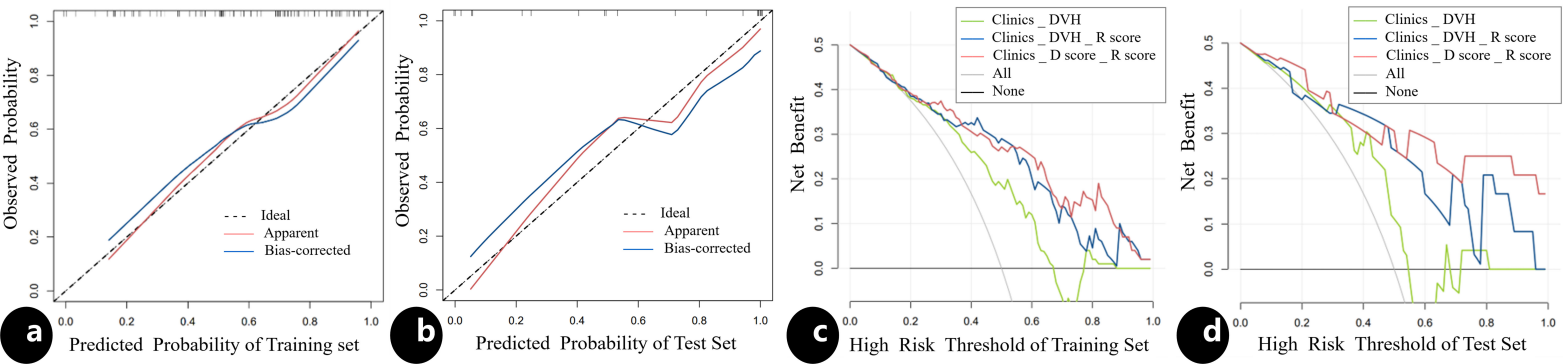
The calibration curve and the decision curve analysis. The calibration curve for the nomogram in the training **(A)** and test **(B)** sets, indicating the goodness of fit of the Clinics+ D-score+ R-score model. The 45° straight line represents the perfect match between the actual (Y-axis) and nomogram-predicted (X-axis) probabilities. A closer distance between two curves indicates higher accuracy; The decision curve analysis for three models in the training **(C)** and test **(D)** sets, the y-axis indicates the net benefit; x-axis indicates threshold probability. The green line, blue line and red line represent net benefit of the Clinics DVH model, the Clinics DVH R-score model and the Clinics+ D-score+ R-score model, respectively. The Clinics+ D-score+ R-score model had a higher overall net benefit in predicting the occurrence of BMS than the other two models and simple diagnoses such as all (gray line) or none (black line) across the full range of threshold probabilities at which a patient would be predicted as BMS.

The results of univariate and multivariate analysis of clinical data were presented in **Table 2**. Multivariable analysis showed that age, PTV volumes and chemotherapy were statistically significant (P < 0.05). Dosimetrics were selected as V10Gy, V20Gy, V30Gy, Dmean of PBM. The nomogram for the Clinics+ D-score+ R-score model was depicted in **Figure 1A**, predicting the probability of BMS, showing an AUC value of 0.751 in the training set. This value surpasses those of the Clinics DVH model (AUC=0.650) and the Clinics DVH R-score model (AUC=0.707). The higher AUC values, the more accurate prediction results. In the test set, the AUC value of the Clinics+ D-score+ R-score model reached 0.743, also exceeding the performance of the Clinics DVH model (AUC=0.638) and the Clinics DVH R-score model (AUC=0.679), as shown in **Figures 1B, C**. The higher the AUC value, the higher the prediction accuracy, so our results show that the dosiomics predictive power was higher than dosimetrics. The calibration curve shows good agreement between the predicted and actual results of the nomogram for the training and the test sets, as shown in **Figures 2A, B**. As is vividly depicted by the DCA curves in **Figures 2C, D**, the Clinics+ D-score+ R-score model had a higher overall net clinical benefit than the other models over most of the range of reasonable threshold probabilities. These results demonstrated that the Clinics+ D-score+ R-score model exhibited superior efficacy in predicting BMS occurrence, and offered a greater net benefit compared to the other models.

**Table 2:** Multifactorial logistic regression analysis of clinical count data in training set patients.

Characteristics	Univariant Logistic Regression Analysis		Multifactorial Logistic Regression Analysis	
	95% *CI* of *OR*	*P* -value	95% *CI* of *OR*	*P* -value
Age	1.042 (1.003~1.082)	0.024	1.050 (1.006~1.096)	0.024
Metastasis	0.748 (0.300~1.865)	0.533	—	—
Chemotherapy	0.375 (0.142~0.987)	0.047	0.293 (0.099~0.868)	0.027
Surgery	2.571 (0.836~7.908)	0.099	—	—
Pathological type	0.492 (0.121~2.005)	0.322	—	—
Radiotherapy techniques	0.762 (0.280~2.075)	0.595	—	—
Total dose	1.080 (0.910~1.282)	0.379	—	—
PTV volumes	1.003 (1.001~1.006)	0.013	1.003 (1.001~1.006)	0.020

## 4 Discussion

This study is the first to demonstrated the feasibility of combining radiomics and dosiomics can predict BMS during radiotherapy for patients with cervical cancer. Previous studies have predominantly concentrated on examining either clinical characteristics or dosimetrics. Compared with the clinical prediction model alone, the prediction performance of the model conbining the radiomics and the dosiomics is greatly improved from CT images and DVH parameters to three-dimensional spatial distribution, and were better predictors of BMS occurrence.

The incidence of severe BMS in 106 cervical cancer patients collected in this study was 64.6%, which aligns with the incidence of BMS grade 3 and above in Han et al.’s study (12). Previous studies have shown that the incidence of BMS is related to chemotherapy regimen (13). However, our study primarily focuses on the impact of radiotherapy dosage on BMS occurrence in patients. Therefore, a detailed investigation into the chemotherapy regimen was not pursued. Concurrent chemotherapy and extended cycles of radiotherapy have been identified as risk factors for acute BMS in patients with cervical cancer following radiotherapy (14, 15). In our study, beyond chemotherapy, age and the volume of the planned target volume (PTV) were also statistically significant (P<0.05) as clinical predictors of BMS. Bone marrow is considered a parallel organ in medical radiobiology, with the incidence of BMS being directly proportional to the administered dose. Previous research indicates that patients with a V10 ≥ 95% and V20 ≥ 76% of pelvic bone marrow exposure are at a higher risk of experiencing Grade 3 or above hematological toxicity (16). Consequently, the same clinical parameters-V10, V20, V30, and Dmean-were incorporated as dosimetric predictors for the occurrence of BMS in this study.

In functional MRI radiomics assessment of pelvic bone marrow changes, Qin et al. identified that the firstorder-Range in FAT IDEAL IQ and firstorder-10Percentile in T2 fat suppression showed obvious dose-response relationship in MR images, reflecting the change of water and fat content at microscopic level during the process of red-yellow bone marrow transformation, indicating the quantitative relationship between bone marrow characteristics and radiomics (17, 18). Compared with CT, MRI has higher sensitivity and specificity in the determination of bone marrow fat, but the magnetic field in MRI-guided radiotherapy has great effects on the dose distribution of radiotherapy, which makes it difficult to design the radiotherapy plan. Therefore, in this study, we used the planning CT images of patients for radiomic features extraction, which was transformed into R-score. The Clinics DVH R-score model (AUC=0.707) was established by combining R-score with clinical and dosimetrics, its accuracy was improved by 0.06 than the Clinics DVH model. This result suggests that the radiomic features of CT also were useful in predicting the occurrence of BMS.

In the same way, the dosiomics is applied to the dose distribution file by using the radiomic method, and the dosiomic features in the three-dimensional space of the dose distribution are extracted, and a model with good prediction performance can be constructed. At present, some studies have indicated that dosiomics is helpful to improve the accuracy of radiation pneumonitis prediction (19, 20). The dosiomics has the ability to predict the side effects of radiotherapy, but there are few studies on the prediction of prognosis of patients with cervical cancer and other pelvic cancers based on dosiomics. So, this is also the innovation of this research. We replaced the dosimetrics with the D-score to establish the prediction model, and found that the prediction accuracy of the model was increased to 0.751. This improvement indicates that the dosiomics is indeed more predictive than the dosimetrics, which was consistent with the previous study.

This study was a single-center study to predict BMS during radiotherapy in patients with cervical cancer. 3 clinical characteristics with predictive value-age, PTV range and chemotherapy-were screen by univariate and multivariate logistic regression. A combined model based on R-score, D-score and 3 clinical characteristics was constructed, and its nomogram was drawn to provide reference for predicting the occurrence of BMS in patients with cervical cancer during radiotherapy. The calibration curve of nomogram shows good agreement between the predicted probabilities of BMS versus actual probabilities of the display nomogram. The results illustrated that the AUC values of the combined model in the training set and the test set were 0.751 and 0.743 respectively, which could better predict the occurrence of BMS.

## 5 Conclusion

The results show that the model established by effective clinical characteristics combined with R-score and D-score has better clinical practicability, and can help physician to identify high-risk patients and provide reference for the design of radiotherapy plan.

There exist some shortcomings in this study. Firstly, this study is a single-center retrospective study with a small sample size and lack of prospective data. Secondly, whether chemotherapy regimens have an impact on BMS development was not further explored in the study. In the future, the authors intend to carry out verification in different centers to improve the prediction performance of the model.

## Data Availability

The raw data supporting the conclusions of this article will be made available by the authors, without undue reservation.
